# Alterations in Methionine Cycle and Wnt/MAPK Signaling Associated with HMBi-Induced Cashmere Growth in Goats

**DOI:** 10.3390/ijms26041663

**Published:** 2025-02-15

**Authors:** Minjie Xi, Jiali Jiang, Bo Wang, Yihan Wang, Meiqi Di, Yuyan Cong, Ruiyang Zhang

**Affiliations:** College of Animal Science and Veterinary Medicine, Shenyang Agricultural University, Shenyang 110866, China; 18804036542@163.com (M.X.); j130654141177@163.com (J.J.); wangbosyau@163.com (B.W.); 15084175610@163.com (Y.W.); dmq13052614001@163.com (M.D.); cyy66@163.com (Y.C.)

**Keywords:** cashmere goats, HMBi supplementation, metabolome, skin tissue, transcriptome

## Abstract

Methionine (Met) was the first limiting amino acid identified in cashmere goats, and 2-hydroxy-4-(methylthio) butanoic acid isopropyl ester (HMBi) can effectively provide Met and encourage cashmere growth in goats. However, existing studies have primarily centered on the trait of cashmere growth and have not delved into the underlying molecular and physiological mechanisms by which HMBi promotes cashmere growth in goats. In the present study, we combined metabolomic and transcriptomic approaches to reveal the effects of HMBi supplementation and its impact on the gene expressions and metabolic profiles within the skin tissue of Liaoning cashmere goats. A total of 14 female Liaoning cashmere goats were randomly allocated to the control (CON) and HMBi groups. The CON group received a basal diet, and the HMBi group was fed the basal diet plus 1.27% HMBi. Our results show that HMBi supplementation significantly increased (*p* < 0.05) the cashmere length and decreased the cashmere diameter in the goats. The metabolomics results show that the HMBi supplementation increased (variable importance in projection >1 and *p* < 0.05) the concentrations of Met, 2-Hydroxy-4-methylthiobutanoic acid (HMB), proline betaine, and 10-hydroxydecanoic acid in the skin tissue of the goats. For HMB degradation and Met cycle-related genes, compared with the CON diets, the HMBi diets elevated (*p* < 0.05) LDHD, MAT1A, and AHCY by 86.33%, 154.54%, and 147.89% in the skin tissue, respectively. Regarding genes related to cell proliferation and differentiation, the HMBi supplementation increased (*p* < 0.05) CCND1, CDK4, IVL, and BMP4 by 113.31%, 107.93%, 291.33%, and 186.21%, respectively. The results of the transcriptome evaluation show that the differential expression genes were mainly enriched (*p* < 0.05) in the Wnt and MAPK signaling pathways. In summary, these findings indicate that the Met cycle, Wnt, and MAPK play important roles in the process of HMBi, promoting cashmere growth in Liaoning cashmere goats.

## 1. Introduction

Liaoning cashmere goats mainly grow in Northeast China and have received significant attention due to their high cashmere yield and cashmere quality [1]. Currently, promoting cashmere growth through nutritional strategies has become a high-priority issue for enhancing the economic benefits of cashmere production. Methionine (Met) is identified as the first - limiting amino acid in cashmere goats, plays a vital role in promoting protein synthesis and follicle development during cashmere growth [2]. However, the degradation of Met in feeds by rumen microbiota significantly reduce the amount of Met reaching the small intestine, resulting in a net decrease in Met utilization [3]. To circumvent the above issue, rumen-protected methionine (RPM) has been widely used to increase the intestinal uptake of Met by avoiding rumen microbiological breakdown [4,5,6,7].

2-hydroxy-4-(methylthio) butanoic acid isopropyl ester (HMBi) is a unique and effective RPM that is currently widely used in dairy cow production [8]. Numerous studies have confirmed that HMBi can improve the growth performance of beef cows and increase the milk yield, milk fat rate, and nitrogen utilization of dairy cows [9,10,11]. However, research on the effects of HMBi treatment in cashmere goats is scarce. One study demonstrates that HMBi supplementation increased the contents of sulfur, cysteine (Cys), and Met in cashmere fibers, thereby promoting cashmere growth [12]. However, previous studies have focused only on the trait of cashmere growth and have not evaluated the mechanism by which HMBi promotes it. Today, our insights into the underlying mechanisms associated with phenotypic characteristics have been deepened by the advancement in omics techniques. Therefore, our study used multi-omics approaches (metabolomics and transcriptomics) to reveal the effects of HMBi on the metabolic profiles and skin tissue expression patterns of Liaoning cashmere goats. Our study provides a theoretical foundation for using HMBi in cashmere goats and reveals the molecular mechanism by which it promotes cashmere growth in Liaoning goats.

## 2. Results

### 2.1. Growth and Cashmere Performance of Goats

As shown in Table S3, the HMBi diets resulted in a greater (*p* < 0.05) cashmere length (23.59 ± 0.14 μm vs. 20.88 ± 0.02 μm) and average daily growth rate of cashmere (0.39 ± 0.000 mm/d vs. 0.35 ± 0.002 mm/d), as well as a smaller (*p* < 0.05) cashmere diameter (16.37 ± 0.53 μm vs. 17.66 ± 0.22 μm) than the CON diet.

### 2.2. Metabolic Profiles of the Skin Tissue of Goats in the CON and HMBi Groups

**Figure 1:**
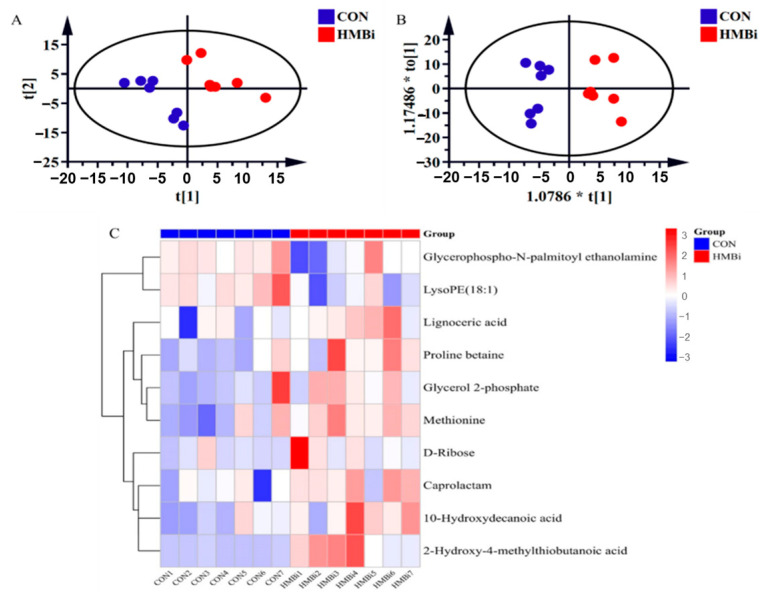
The effects of HMBi supplementation on the metabolites in the skin tissue of cashmere goats. (**A**) Partial least squares discriminant analysis (PLS-DA) and (**B**) orthogonal partial least squares discriminant analysis (OPLS-DA) of skin metabolites identified using the metabolome approach. (**C**) The differential metabolites in the skin tissue between the CON and HMBi groups. Only the metabolites that met the criteria of a variable importance in projection (VIP) > 1 and *p* < 0.05 were defined as differential metabolites. CON, control group; HMBi, 2-hydroxy-4-(methylthio) butanoic acid isopropyl ester supplementation group.

To reveal the effects of HMBi on the metabolic profiles of the skin tissue of the goats, we utilized liquid chromatography–mass spectrometry (LC-MS)-based metabolic approaches. The results of the partial least squares discriminant analysis (PLS-DA), as shown in Figure 1A, and the orthogonal partial least square discriminant analysis (OPLS-DA) analysis, as presented in Figure 1B, show that the metabolites of the CON and HMBi diets are clearly distributed in different areas in the plot, without any overlap. Moreover, we defined the metabolites as differential metabolites in skin tissue based on the criteria of a variable importance in projection (VIP) > 1 and *p* < 0.05. The results (Figure 1C) show that, in comparison to the CON group, the HMBi group exhibited a significant increase (*p* < 0.05) in the concentrations of caprolactam, Met, lignoceric acid, proline betaine, 10-hydroxydecanoic acid, D-Ribose, glycerol 2-phosphate, and 2-hydroxy-4-methyl-thio butanoic acid (HMB). The HMBi group also displayed a noteworthy decline (*p* < 0.05) in the concentrations of glycerophospho-N-palmitoyl ethanolamine and LysoPE (18:1) in the skin tissue of goats.

### 2.3. Met Metabolism in the Skin Tissue of Goats in the CON and HMBi Groups

**Figure 2:**
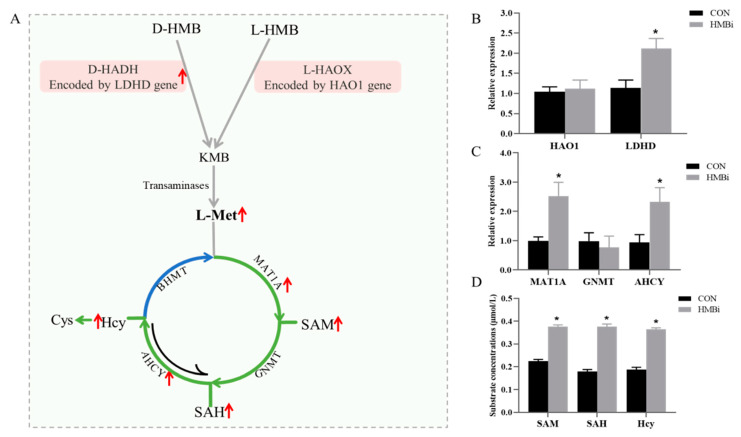
The effects of HMBi supplementation on the HMB degradation and methionine cycle metabolism in the skin tissue of cashmere goats. (**A**) Metabolic pathway diagram of HMB degradation; (**B**) Relative expression of HAO1 and LDHD genes; (**C**) Relative expression of MAT1A, GNMT and AHCY genes; (**D**) Concentrations of SAM, SAH and Hcy substrates. CON, control group; HMBi, 2-hydroxy-4-(methylthio) butanoic acid isopropyl ester supplementation group. HMB, 2-hydroxy-4-(methylthio) butanoic acid; D-HADH, D-2-hydroxy acid dehydrogenase; L-HAOX, L-2-hydroxy acid oxidase; KMB, 2-keto-4-(methylthio) butanoic acid; *MAT1A, methionine adenosyltransferase 1A; GNMT, glycine N-methyltransferase; AHCY, adenosylhomocysteinase; BHMT, homocysteine methyltransferase;* SAM, methionine adenosyltransferase; SAH, S-adenosyl homocysteine; Hcy, homocysteic acid; Cys, cysteine; *, *p* < 0.05; red, incresed.

We used real-time quantitative PCR (qRT-PCR) to detect the mRNA expression of HMB degradation- and Met cycle metabolism-related genes (Figure 2A). Our results document that (Figure 2B), in comparison to the CON group, the HMBi group had an increased (*p* < 0.05) expression level of *L-lactate dehydrogenase D (LDHD)* by 86.33% in the skin tissue of the goats. However, the dietary treatments had no effect on the mRNA expression of *hxydroxyacid oxidase 1 (HAO1)* in the skin tissue (*p* > 0.05). Meanwhile, our results (Figure 2C) also indicate that HMBi supplementation significantly increased (*p* < 0.05) the mRNA expressions of *methionine adenosyltransferase 1A (MAT1A)* and *adenosylhomocysteinase (AHCY)* by 154.54% and 147.89%, respectively. However, the dietary treatments had no significant effect on the mRNA expression of *glycine N-methyltransferase (GNMT)* in the skin tissue (*p* > 0.05). Finally, the results of the ELISA show that (Figure 2D) the contents of methionine adenosyltransferase (SAM), S-adenosyl homocysteine (SAH), and homocysteic acid (Hcy) in the skin tissue of the goats in the HMBi group were notably greater (*p* < 0.05) than those in the CON group.

### 2.4. Gene Changes Related to Cell Proliferation and Differentiation

**Figure 3:**
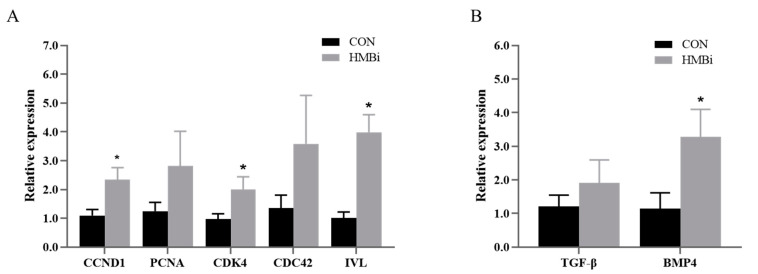
Th effects of HMBi supplementation on cell proliferation (**A**) and differentiation (**B**) genes in the skin tissue of cashmere goats. CON, control group; HMBi, 2-hydroxy-4-(methylthio) butanoic acid isopropyl ester supplementation group; *CCND1, cyclin D1; PCNA, proliferating cell nuclear antigen; CDK4, cyclin dependent kinase 4; CDC42, cell division cycle 42; IVL, involucrin; TGF-β, transforming growth factor-β; BMP4, bone morphogenetic protein 4*; *, *p* < 0.05.

The growth of cashmere is inseparable from cell proliferation and differentiation. Therefore, we conducted qRT-PCR analysis of related genes. The findings show that (Figure 3A), compared with the CON group, the HMBi group had significantly increased (*p* < 0.05) relative expression levels of genes *cyclin D1 (CCND1), cyclin dependent kinase 4 (CDK4),* and *involucrin (IVL)* by 113.31%, 107.93%, and 291.33%, respectively. Regarding the genes related to cell differentiation (Figure 3B), the relative expression of *bone morphogenetic protein 4 (BMP4)* was notably higher (186.21%) in the HMBi group than that in the CON group (*p* < 0.05). Moreover, there were no significant differences in the relative expression of *proliferating cell nuclear antigen (PCNA), cell division cycle 42 (CDC42)*, or *transforming growth factor-β (TGF-β)* in the skin of the cashmere goats (*p* > 0.05).

### 2.5. Functional Analysis of Differential Expression Genes (DEGs) Detected in the CON and HMBi Diets

**Figure 4:**
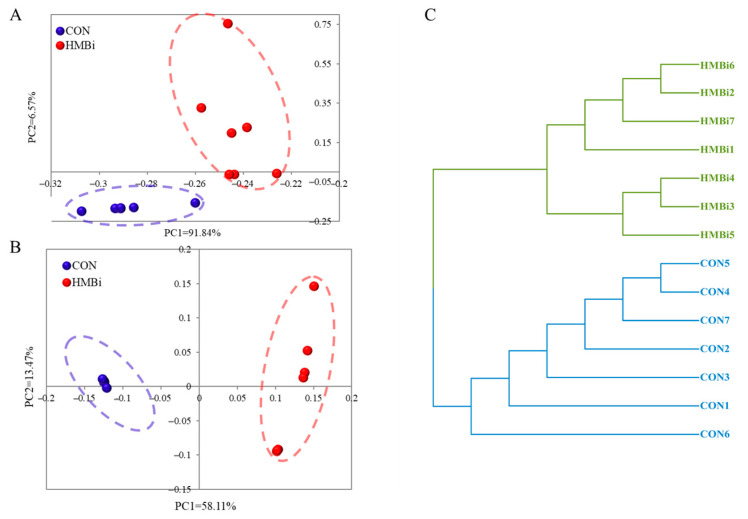
(**A**) Principal component analysis (PCA); (**B**) principal coordinate analysis (PCoA); and (**C**) cluster analysis of transcriptome-based gene expressions in the skin tissue of goats fed the CON and HMBi diets. CON, control diet; HMBi, 2-hydroxy-4-(methylthio) butanoic acid isopropyl ester diet.

We used principal component analysis (PCA) and principal coordinates analysis (PCoA) to evaluate the overall effects of HMBi on gene expression in the skin tissue of goats. Our results show, in both the PCA (Figure 4A) and PCoA plots (Figure 4B), that the general gene expressions of the samples from the two treatment groups are clustered separately and distinctly. Correspondingly, the results of the sample cluster analysis (Figure 4C) demonstrate that the samples from the same group are grouped together, while those from the CON and HMBi are clustered into distinct branches.

**Figure 5:**
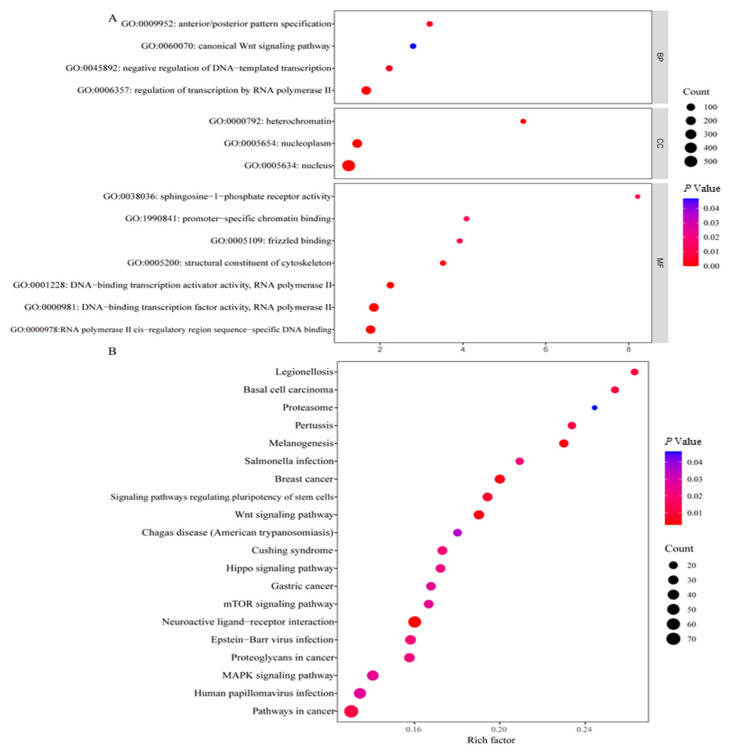
Enrichment analysis results of Gene Ontology (GO) terms (**A**) and Kyoto Encyclopedia of Genes and Genomes (KEGG) pathways (**B**).

In the present study, we identified 2143 genes that were significantly differentially expressed in the goats fed the HMBi diets compared with those fed the CON diets, including 1749 upregulated and 394 downregulated DEGs (Figure S1). To further analyze the DEGs, GO and KEGG enrichment analyses were performed. The enrichment analysis of GO terms showed that (Figure S2), at a higher classification level, these DEGs were significantly enriched in (*p* < 0.05) the regulation of biological processes, such as anatomical structure development, response to stress, cellular developmental process, and cell population proliferation. The results also show that (Figure 5A), at a lower classification level, DEGs were markedly enriched in (*p* < 0.05) the following GO terms in biological processes: regulation of transcription by RNA polymerase II, negative regulation of DNA-templated transcription, canonical Wnt signaling pathway, and anterior/posterior pattern specification. The enrichment analysis of the KEGG pathways showed that (Figure 5B) DEGs were significantly (*p* < 0.05) enriched in the MAPK signaling pathway, neuroactive ligand–receptor interactions, the Wnt signaling pathway, melanogenesis, and other several pathways.

### 2.6. DEGs in Wnt Signaling Pathway

**Figure 6:**
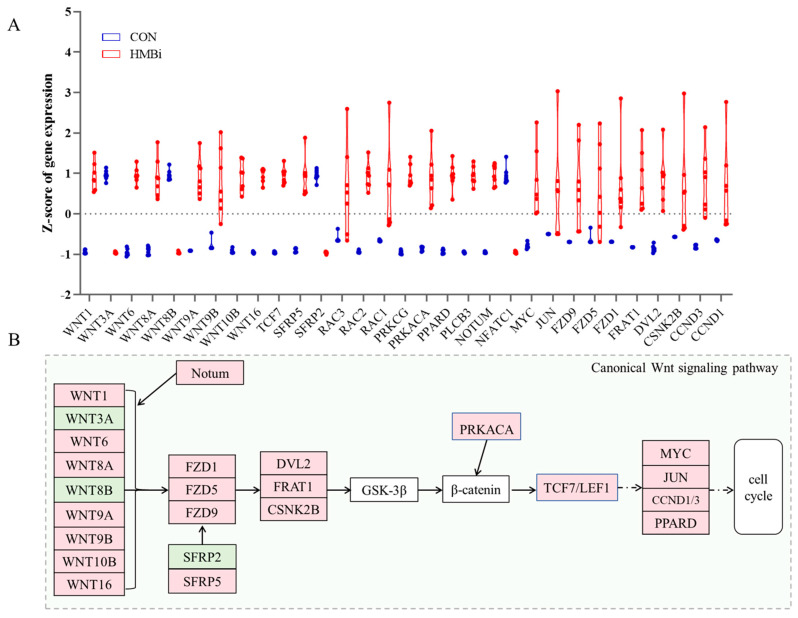
HMBi supplementation activated the Wnt signaling pathway in the skin tissue of cashmere goats. (**A**) DEGs in the Wnt signaling pathway affected by dietary treatments. CON, control group; HMBi, 2-hydroxy-4-(methylthio) butanoic acid isopropyl ester group. (**B**) Schematic plot of canonical Wnt signaling pathway. Light red, upregulated; light green, downregulated; white, unchanged. WNT, Wnt family member; *SFRP, secreted frizzled related protein; TCF7, transcription factor 7; CCND1, cyclin D1*.

Among the enriched KEGG pathways, the Wnt signaling pathway attracted our attention. Our results of further analysis revealed that (Figure 6A) a total of 31 DEGs in this pathway were identified between the CON and HMBi groups. In general, except for the gene *nuclear factor of activated T cells 1 (NFATC1)*, *secreted frizzled related protein 2 (SFRP2), Wnt family member (WNT) 3A, and WNT8B*, the remaining DEGs, such as *WNT1, WNT9, WNT10B, transcription factor 7 (TCF7), and CCND1*, were substantially upregulated (*p* < 0.05) in the HMBi group (Figure 6A). Moreover, the enrichment analysis indicated that the canonical Wnt signaling pathway was significantly affected (*p* < 0.05) by the dietary treatments in the present study. Hence, we developed a schematic diagram of the canonical Wnt signaling pathway (Figure 6B), and the results show that most of the genes related to this pathway were significantly upregulated (*p* < 0.05) after HMBi supplementation.

**Figure 7:**
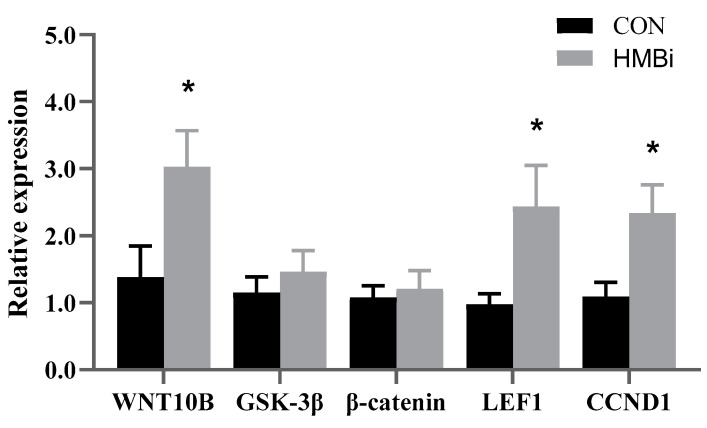
The expressions of key genes in the Wnt signaling pathway measured by the qRT-PCR method. CON, control group; HMBi, 2-hydroxy-4-(methylthio) butanoic acid isopropyl ester group; WNT, WNT family member; GSK-3β, Glycogen Synthase Kinase-3β; LEF1, lymphoid enhancer binding factor 1. *, *p* < 0.05.

Next, we used qRT-PCR to determine the key genes in the Wnt signaling pathway and validate the accuracy of the gene expressions harvested from the transcriptome. The results show that (Figure 7), compared with CON group, the HMBi group significantly increased (*p* < 0.05) the relative expression of WNT10B, *lymphoid enhancer binding factor 1 (LEF1)*, and *CCND1* by 119.36%, 149.29%, and 113.31%, respectively. Moreover, the dietary treatment had no effect on the genes *Glycogen Synthase Kinase-3β (GSK-3β) and β-catenin* in the skin of goats (*p* > 0.05). The changes in the aforementioned genes are congruent with the findings of the transcriptome sequencing.

### 2.7. DEGs in MAPK Signaling Pathway

**Figure 8:**
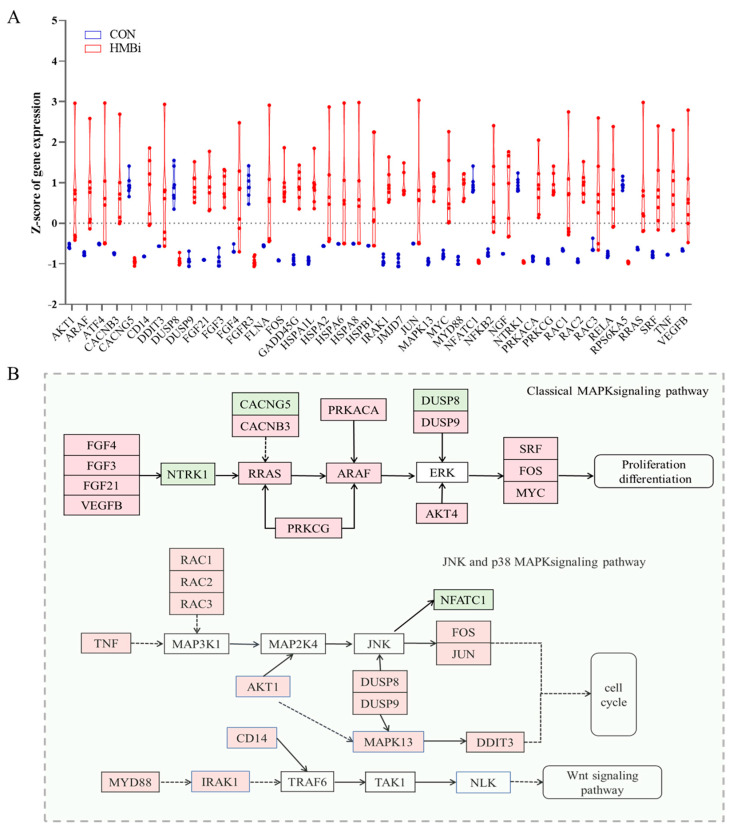
HMBi supplementation activated the MAPK signaling pathway in the skin tissue of cashmere goats. (**A**) DEGs in the MAPK signaling pathway affected by dietary treatments. CON, control group; HMBi, 2-hydroxy-4-(methylthio) butanoic acid isopropyl ester group. (**B**) Schematic plot of the MAPK signaling pathway. Light red, regulated; light green, downregulated; white, unchanged.

Among these enriched KEGG pathways, in addition to the Wnt signaling pathway, the MAPK signaling pathway was also related to cashmere growth. Our further analysis showed that (Figure 8A) a total of 42 DEGs in this pathway were identified between the CON and HMBi groups. As shown in Figure 8A,B, the HMBi group significantly upregulated (*p* < 0.05) the expressions of *AKT serine/threonine kinase 1* (*AKT1*), *Fibroblast growth factor 21* (*FGF21*), *related RAS viral* (*rras*) *oncogene homolog (RRAS), protein kinase C gamma (PRKCG)*, *Fos proto-oncogene* (*FOS*)*, Jun proto-oncogene* (*JUN*), *vascular endothelial growth factor B* (*VEGFB*), *and mitogen-activated protein kinase 13* (*MAPK13*). In contrast, the expressions of *calcium voltage-gated channel auxiliary subunit gamma 5* (*CACNG5*), *NFATC1*, and *ribosomal protein S6 kinase A5* (*RPS6KA5*) were significantly downregulated (*p* < 0.05) in the HMBi group.

## 3. Discussion

Given the current relatively high breeding level, promoting cashmere growth in cashmere goats through nutritional strategies has become a priority for enhancing the economic benefits of cashmere production [13]. Met is the first identified limiting amino acid and performs a direct task in protein synthesis, which encourages cashmere growth and development [2]. Our results show that dietary HMBi supplementation significantly increased the cashmere length and reduced the cashmere growth diameter, indicating that HMBi had the effect of promoting cashmere growth and enhancing cashmere performance in goats. These results are consistent with the findings of Li et al. [14]. Based on the above results, we further conducted our study to explore the underlying molecular mechanisms by which HMBi promotes cashmere growth in goats.

### 3.1. HMBi Supplementation Altered the Metabolism Pattern and Increased the HMB and Met Reaching the Skin Tissue of Goats

Our LC-MS results indicate that HMBi supplementation significantly increased the contents of D-Ribose and 10-hydroxydecanoic acid in the skin tissue of the goats in the present study. D-Ribose, a component of essential biomolecules, such as adenine nucleotides, riboflavin, and RNA, also serves as a substrate in ATP synthesis and enhances cellular mitochondrial function [15,16]. Consequently, the increased content of D-Ribose in the HMBi group may have boosted ATP metabolism, accelerating biological reactions, such as protein synthesis and cell proliferation [15]. Additionally, 10-hydroxydecanoic acid was reported to be an anti-inflammatory fatty acid that could inhibit inflammatory responses by modulating proteins in the NF-κB and MAPK signaling pathways [17,18]. Thus, the rise in the contents of D-Ribose and 10-hydroxydecanoic acid in the skin tissue suggest that HMBi supplementation may have helped maintain the immune system and normal functions of goats in the present study.

Moreover, our results suggest that HMBi supplementation increased the contents of Met and HMB in the skin tissue of goats. HMBi can be rapidly degraded in the rumen and absorbed in the form of HMB, which is then converted into Met in the liver and other tissues [19]. The present study detected HMB in the skin of goats using LC-MS, confirming the aforementioned viewpoint. To our knowledge, this is the first report of the presence of HMB in the skin tissue of cashmere goats following dietary HMBi supplementation. Additionally, Met is the first identified limiting amino acid in ruminants and plays a vital role in enhancing skin protein synthesis and wool growth in animals [2,20]. The introduction of Met in vitro was shown to increase the number of hair follicles on the dorsal surface of Rex rabbit, as well as promote hair shaft growth in vivo [21]. Therefore, we reasonably speculate that the increased contents of HMB and Met were the primary reasons for the accelerated cashmere growth after supplementing with HMBi in the diets of goats. Furthermore, our results indicate that the HMBi diets had an increased content of proline betaine in the skin tissue of the goats. Previous studies have confirmed that proline betaine alleviates inflammation and inhibits cell apoptosis [22], and it acts as an inhibitor of homocysteine methyltransferase (BHMT), which affects the generation of Hcy [23,24]. Hcy can be converted into Met under the catalytic action of BHMT, making this step a vital part of the Met cycle pathway [25]. The skin tissue and hair follicles of fur-bearing animals depend on Met metabolism to convert the Met into Cys, thereby meeting the requirements for hair growth [20]. During Met metabolism, the Met cycle serves as a crucial pathway. Hence, the above results suggest that HMBi supplementation may affect Met cycle metabolism in the skin tissue of goats.

### 3.2. Met Metabolism in the Skin Tissue of Goats in the CON and HMBi Groups

According to the above results, we investigated the degradation process of HMB into Met and the subsequent Met cycle in the skin tissue of goats. Our findings indicate that HMBi supplementation increased the expression of *LDHD* gene in the skin tissue. HMB exists in two isomeric forms, D-HMB and L-HMB, which are oxidized to 2-keto-4 (methylthio) butanoic acid (KMB) by *LDHD* and *HAO1*, respectively [26]. Thus, the increased expression of the *LDHD* gene suggests that HMBi supplementation promoted the conversion of D-HMB into KMB, while having no effect on the conversion of L-HMB into KMB in the skin tissue of the goats in the present study.

KMB is converted into Met through the action of transaminases [27], subsequently undergoing Met metabolism to exert its physiological effects. Met is catabolized into Hcy via the Met cycle, and Hcy can then be further catabolized to form Cys after transsulfuration [20]. Cys is a fundamental amino acid for synthesizing cashmere fibers and directly affects hair follicle function, influencing cashmere growth [28,29]. Our results regarding key genes and substrates in the Met cycle indicate that HMBi supplementation enhanced the mRNA expression of *MAT1A* and *AHCY* and increased the contents of SAM, SAH, and Hcy in the skin tissue of the goats. Thus, these results confirm that HMBi supplementation promotes the Met cycle, resulting in the accumulation of more Hcy in the skin tissue of the goats in the present study. Moreover, a previous study confirmed that the Hcy generation mediated by the Met cycle was a key rate-limiting step in regulating Cys generation [30]. As mentioned above, higher proline betaine levels had an inhibitory effect on the BHMT enzyme involved in the conversion of Hcy into Met. Hence, the above results imply that the promotion by HMBi for the conversion of Met to Cys is one of the reasons for cashmere growth.

### 3.3. HMBi Supplementation Promoted the Cell Proliferation and Differentiation in the Skin Tissue of Goats

Cashmere growth is closely linked to the processes of cell proliferation and differentiation. Therefore, we assessed the mRNA expressions of genes associated with these processes. Our results indicate that HMBi supplementation elevated the expressions of *CCND1, CDK4, IVL,* and *BMP4* in the skin tissue of the goats in the present study. *CCND1* and *CDK4* are important components of the cell cycle regulatory mechanism, and an elevation in the levels of the *CCND1-CDK4* complex promotes cell proliferation and shortens the cell proliferation cycle [31,32]. Moreover, *IVL* is often employed as an indicator of keratinocyte differentiation, as it serves as a substrate for keratinocyte glutamine transferase and scaffolds the creation of the epidermal keratinization membrane [33]. Consequently, the increased expressions of *CCND1*, *CDK4* and *IVL* suggest that the HMBi diets promoted cell proliferation and differentiation in the skin tissue of the goats in the present study. In addition, BMP signaling plays a role in epidermal development by regulating hair follicle initiation and differentiation during skin morphogenesis [34,35]. The proliferation and migration of root sheath cells outside of the hair follicle have been shown to be facilitated by *BMP4*, which is mostly expressed in mature hair papilla cells [36]. Thus, the above results confirm that HMBi supplementation promoted the cell proliferation, differentiation, and hair follicle development at the molecular level in the skin tissue of cashmere goats.

### 3.4. Cashmere Growth After HMBi Supplementation Was Related to the Wnt and MAPK Signaling Pathways

Cashmere growth is a highly complex process regulated by multiple signaling pathways. For instance, the Wnt signaling pathway, FGF signaling pathway, and BMP signaling pathway have been shown to be crucial in controlling the expression of genes associated with hair follicle development in mammals [37,38]. Among these, Wnt signaling is considered to be the main regulator of the morphogenesis process, primarily responsible for initiating hair follicle formation [39]. In the present study, our enrichment analyses of GO and KEGG indicate that the DEGs were notably enriched in the Wnt signaling, with most genes involved in this signaling being upregulated. This suggests that Wnt signaling plays an important role in promoting cashmere growth through HMBi supplementation. Specifically, our results reveal that the expressions of *Wnt10B* and *LEF1* in the skin tissue of the goats was notably greater in the HMBi group, while *GSK-3β* and *β-catenin* exhibited no discernible trend. *Wnt10B* was predominantly expressed in hair follicle stem cells, pre-cortical hair cells, and stromal cells [40], and it has been shown to significantly enhance epithelial cell differentiation and hair shaft growth [41]. *LEF1* interacts with *β-catenin* to regulate cell proliferation and differentiation, facilitating the delivery of *β-catenin* to target cells [42,43]. Met supplementation in diets has been shown to dramatically upregulate the *Wnt10B* and *LEF1* genes, reduce damage to hair follicles, and increase the density of hair follicles in the dorsal skin of Rax rabbits [44]. Additionally, a prior study showed that RPM supplementation increased the expression of genes related to fetal hair follicle development, including *Wnt10B*, *Wnt3a*, and *BMP4* in cashmere goats [45]. Therefore, our findings imply that HMBi supplementation stimulated the proliferation and differentiation of hair follicle cells by activating Wnt signaling.

In addition, the MAPK pathway regulates many vital physiological functions, including differentiation, cell division, adaptation to environmental stress, and inflammation [46,47]. Moreover, studies have demonstrated that the MAPK and Wnt signaling pathways could collaborate to enhance cell differentiation and proliferation [48]. Our results also show that the DEGs were substantially enriched in the MAPK pathway in the skin tissue of the goats following HMBi supplementation. Specifically, our results reveal that the gene expressions of *AKT1, FGF21, RRAS, FOS, JUN,* and *VEGFB* in the skin tissue of the goats were markedly higher in the HMBi group compared to the CON group. The P13/AKT pathway has been reported to regulate fibroblasts proliferation, promote faster skin wound healing, and contribute to the proliferation of hair follicle stem cells [49,50]. Moreover, research has shown that an AKT1-mediated post-transcriptional mechanism was involved in promoting the release of *VEGFB* in keratinocyte-forming cells during skin wound healing [51]. The growth cycle of hair follicles is significantly influenced by VEGF, which promotes follicular angiogenesis, hair renewal, and an increase in the size of the hair follicle shaft [52]. VEGF was found to promote the multiplication of outer root sheath cells within the secondary hair follicles of cashmere goats [53]. Furthermore, experimental evidence has shown that *FGF21* stimulated epidermal cell migration and differentiation, thereby accelerating the healing process [54]. *FGF21* was also proven to enhance fibroblast migration by activating the AKT protein and the Wnt signaling pathway [55]. Hence, the elevated expressions of genes within MAPK signaling substantiate the proposition that the HMBi diets stimulated hair follicle cell proliferation in cashmere goats, thereby accelerating cashmere growth.

It is worth noting that, as mentioned earlier, the MAPK and Wnt signaling pathways could collaborate to enhance cell differentiation and proliferation [48]. Numerous studies have confirmed that there is complex crosstalk between the MAPK and Wnt signaling pathways, with each regulating the other [56]. For example, phosphoinositide-3 kinase (PI3K)/AKt signaling could impact the activation of the Wnt pathway by phosphorylating *GSK-3β* at a specific site [50]. However, the role of this crosstalk between the MAPK and Wnt signaling pathways in the process of HMBi promoting cashmere growth deserves further study.

## 4. Materials and Methods

### 4.1. Animals Experiment and Diets

Fourteen female and healthy Liaoning cashmere goats of similar age (eight months) and comparable body weight (averaging 25.63 ± 0.87 kg) were randomly assigned to either the experimental (HMBi) or control (CON) group, with seven animals in each. The control group was provided a basal diet, while the treatment group was provided a basal diet plus 1.27% HMBi (MetaSmart™, Adisseo, Alpharetta, GA, USA). The experiment was designed to include a 7-day pre-feeding period, followed by a 60-day formal experiment. All of the animals were housed in individual pens and had free access to feeds and water. The ingredient and nutrient composition of the basal diet is presented in Table S1. During the experiment, the cashmere length, growth rate, and diameter were measured.

### 4.2. Sample Collection

Upon completion of the formal experiment, 4 h after the morning feeding, all experimental goats were euthanized and slaughtered. After each goat’s scapular region was sampled, a 1 cm × 1 cm skin biopsy was collected and immediately stored in liquid nitrogen. Subsequently, the collected samples were immediately placed in storage at −80 °C.

### 4.3. Liquid Chromatography–Mass Spectrometry (LC-MS) Analysis

After thawing and thoroughly mixing the sample, 50 mg of the skin sample was combined with 800 mL of methanol. The mixture was homogenized for 180 s at 65 Hz using a high-speed tissue homogenizer (Tissuelyser II, Qiagen, Hilden, Germany). This was followed by 30 min of sonication at 4 °C in a sonicator bath (SB–5200DTDN, Ningbo Scientz Biotechnology Co., Ltd., Ningbo, China). After stewing, the mixture was centrifuged (Eppendorf centrifuge 5424R, Eppendorf GMBH, Hamburg, Germany) for 15 min at 12,000× *g* at 4 °C. Following stewing, it was centrifuged for 15 min at 12,000× *g* at 4 °C. After collecting and reboiling the supernatant, it was centrifuged for 15 min at 12,000× *g* at 4 °C. The analysis was conducted using a Waters ACQUITY UPLC^®^ system equipped with an ACQUITY UPLC^®^ HSS T3 column (length: 100 mm, diameter: 2.1 mm, particle size: 1.8 μm; Waters Corp., Milford, MA, USA). The flow rate of the instrument was set at 0.3 mL/min, and the column temperature was maintained at 40 °C. The sample injection volume was 5 μL. The metabolomic data were analyzed and annotated using Compound Discoverer 3.3 software (Thermo Fisher Scientific, Waltham, MA, USA). Subsequently, the normalized peaks of the metabolites were input into SIMCA-P+ 14.1 software (Umetrics, Umea, Sweden) to perform PLS-DA and OPLS-DA. During the PLS-DA, the variable importance in projection (VIP) values were calculated.

### 4.4. Substrates of Met Cycle Measured by ELISA

In the present study, enzyme-linked immunosorbent test (ELISA) technology was used to measure the substrates of the Met cycle. In the present study, the Goat SAM ELISA Kit (BS-E16189O2, Bo Shen, Nanjing, Jiangsu, China), Goat SAH ELISA Kit (BS-E16190O2, Bo Shen, Jiangsu, China), and Goat Hcy ELISA Kit (BS-E19644O2, Bo Shen, Jiangsu, China) were adopted to determine the concentrations of SAM, SAH and Hcy. The kits’ precoated wells were filled one after the other with the specimens, standards, and horseradish peroxidase (HRP)-labeled detection antibodies. The HRP-labeled detection antibody was added after a thorough cleaning procedure and a preheating interval. The substrate TMB, which was first turned blue by peroxidase catalysis and then yellow by acid, was used to create the color. The concentrations of SAM, SAH, and Hcy in the sample were correlated with the color intensity. A F50 fully automated ELISA analyzer (Tecan Group Ltd., Männedorf, Switzerland) was used to measure the absorbance (OD) at 450 nm and compute the sample concentrations.

### 4.5. Extraction of the Skin RNA and qRT-PCR

Approximately 200 mg of skin tissue samples were homogenized with 1 mL of TRIzol^®^ Reagent (Takara Bio, Otsu, Japan) to isolate the total RNA. The integrity and concentration of RNA were assessed using 2100 Bioanalyser (Agilent, Santa Clara, CA, USA) and ND-2000 (NanoDrop Technologies, Thermo Fisher Scientific, USA). High-quality RNA samples (with OD260/280 values between 1.8 and 2.2 and OD260/230 values no less than 2.0) were employed for subsequent qRT-PCR and transcriptome analysis.

Thereafter, a total of 1 µg of RNA sample was employed for the reverse transcription process, utilizing a PrimeScript^®^ RT reagent kit along with a gDNA Eraser (Takara Bio, Otsu, Japan). Three replicates were set up for the PCR reaction, and PCR amplification was conducted on a Roche LightCycler^®^ 96 system (Roche Diagnostics, Basel, Switzerland). To normalize the mRNA expressions of the target genes, β-actin was adopted as a housekeeping gene [57], and the relative expressions of each gene were calculated using the 2-(ΔΔCt) method [58]. All the primers applied in this current study are presented in Table S2.

### 4.6. Library Preparation and Illumina HiSeq Sequencing

RNA-seq transcriptome libraries were created using the TruSeqTM RNA Sample Preparation Kit from Illumina (San Diego, CA, USA). In short, fragmentation buffer was used to break down the mRNA after it was extracted using polyA selection on oligo(dT) beads. Following Illumina’s procedure, the processes of cDNA synthesis, A-base addition, and adaptor ligations were performed. After that, libraries were sized for cDNA target fragments, and phusion DNA Polymerase was then used to amplify the libraries for 15 PCR cycles. After TBS380 quantification, paired-end libraries were sequenced by Illumina NovaSeq 6000 sequencing (BIOZERON Co., Ltd., Shanghai, China)

### 4.7. Reads Quality Control and Mapping

Raw paired-end reads were trimmed and quality-controlled using Trimmomatic (v0.36, http://www.usadellab.org/cms/index.php?page=trimmomatic) (accessed on 15 October 2024). Then, clean reads were individually aligned to the reference genome in the orientation mode using hisat2 (https://ccb.jhu.edu/software/hisat2/index.shtml) (accessed on 15 October 2024). This program was utilized using its default settings for mapping. Qualimap v2.2.1 was used to evaluate the quality of the data [59].

### 4.8. Identification of DEGs and Functional Analysis

The fragments per kilobase of exon per million mapped reads (FRKM) approach was applied to determine the expression level of each gene to discover the DEGs between the two distinct samples. The edgeR package (http://www.bioconductor.org/packages/release/bioc/html/edgeR.html/) (accessed on 15 October 2024) in R software (version 4.3.1) was utilized to conduct differential expression analysis. Both the false discovery rate (FDR) and the logarithm of the fold change had to be less than 0.05 for the DEGs between two samples to be chosen. The Kyoto Encyclopedia of Genes and Genomes (KEGG) pathway analysis and Gene Ontology (GO) functional enrichment were executed with KOBAS (http://kobas.cbi.pku.edu.cn/home.do) (accessed on 15 October 2024) and DAVID (https://davidbioinformatics.nih.gov) (accessed on 15 October 2024) in order to comprehend the functionalities of the differentially expressed genes. The DEGs exhibited significant enrichment in the GO terms and KEGG pathways at an adjusted *p*-value below 0.05.

## 5. Conclusions

**Figure 9:**
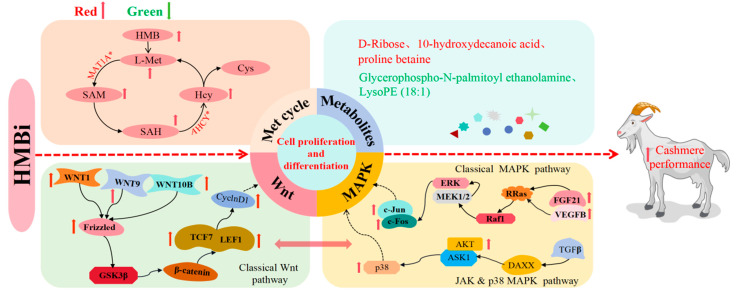
The proposed schematic diagram of the molecular mechanism by which HMBi supplementation promotes the cashmere performance in Liaoning Cashmere goats. HMBi, 2-hydroxy-4-(methylthio) butanoic acid isopropyl ester; HMB, 2-hydroxy-4methyl-thio butanoic acid; SAM, methionine adenosyltransferase; SAH, S-adenosyl homocysteine; Hcy, homocysteic acid; Cys, cysteine; Red, increased; Green, decreased.

In summary, LC-MS-based metabolome and transcriptome analyses were utilized to reveal the molecular mechanism by which HMBi supplementation improves cashmere growth in goats. The proposed schematic diagram of the molecular mechanism in the present study is presented in Figure 9. Overall, our results reveal that HMBi supplementation improved cashmere growth and promoted cell proliferation and differentiation in the skin tissue of goats. The HMBi supplementation increased the contents of HMB and Met in the skin tissue and enhanced the relative mRNA expressions (LDHD, MAT1A, and AHCY) and substrate concentrations (SAM, SAH, and Hcy) related to the degradation of HMB and Met cycle metabolism in the skin tissue of goats. Moreover, the dietary supplementation of HMBi resulted in significant enrichments of differentially expressed genes in the Wnt and MAPK signaling pathways, which are related to cashmere growth. These findings provide new insight into the molecular mechanism by which HMBi improves cashmere growth in cashmere goats. Concurrently, our findings also provide effective nutritional strategies to improve cashmere performance and increase the economic benefits for cashmere goat producers. In the future, hair follicle culture and Western blotting should be conducted to verify the role of the methionine cycle and Wnt/MAPK Signaling in the promotion of cashmere growth by HMBi.

## Data Availability

The data presented in this study are available upon request from the corresponding author.

## Supplementary Materials

The following supporting information can be downloaded at: https://www.mdpi.com/article/10.3390/ijms26041663/s1.

## Abbreviations

The following abbreviations are used in this manuscript:

AKT1	AKT serine/threonine kinase 1
AHCY	Adenosylhomocysteinase
BMP4	Bone morphogenetic protein 4
BHMT	Homocysteine methyltransferase
CDK4	Cyclin-dependent kinase 4
CCND1	Cyclin D1
CDC42	Cell division cycle 42
CACNG5	Calcium voltage-gated channel auxiliary subunit gamma 5
Cys	Cysteine
DEGs	Differential expression genes
ELISA	Enzyme-linked immunosorbent test
FRKM	Fragments per kilobase of exon per million mapped reads
FDR	False discovery rate
FOS	Fos proto-oncogene
FGF21	Fibroblast growth factor 21
GO	Gene Ontology
GNMT	Glycine N-methyltransferase
GSK-3β	Glycogen Synthase Kinase-3β
HMBi	2-hydroxy-4-(methylthio) butanoic acid isopropyl ester
HMB	2-hydroxy-4methyl-thio butanoic acid
Hcy	Homocysteic acid
HRP	Horseradish peroxidase
HAO1	Hxydroxyacid oxidase 1
IVL	Involucrin
JUN	Jun proto-oncogene
KMB	2-keto-4 (methylthio) butanoic acid
KEGG	Kyoto Encyclopedia of Genes and Genomes
LDHD	L–lactate dehydrogenase D
LEF1	Lymphoid enhancer binding factor 1
Met	Methionine
MAT1A	Methionine adenosyltransferase 1A
MAPK13	Mitogen-activated protein kinase 13
NFATC1	Nuclear factor of activated T cells 1
PRKCG	Protein kinase C gamma
PCNA	Proliferating cell nuclear antigen
RPM	Rumen-protected methionine
RRAS	Related RAS viral (r-ras) oncogene homolog
RPS6KA5	Ribosomal protein S6 kinase A5
SAM	Methionine adenosyltransferase
SAH	S-adenosyl homocysteine
SFRP2	Secreted frizzled related protein 2
TGF-β	Transforming growth factor-β
TCF7	Transcription factor 7
VEGFB	Vascular endothelial growth factor B
WNT	Wnt family member
