# Supplementary material for: Alterations in Methionine Cycle and Wnt/MAPK Signaling Associated with HMBi-Induced Cashmere Growth in Goats
Source: Int J Mol Sci. 2025 Feb 15;26(4):1663. doi: 10.3390/ijms26041663 (PMC11855240; doi:10.3390/ijms26041663)

Table S1 The ingredients and nutrient composition of the basal diet.

Ingredient	(%)	Nutrient composition[1]	
Alfafa	25	ME（MJ/Kg)	8.67
Peanut vine	45	CP（%）	11.89
Corn	18	Ca（%）	0.88
Soybean meal	6	P（%）	0.34
Wheat bran	4.5	NDF（%）	43.95
Salt	0.5	ADF（%）	28.37
Dicalcium phosphate	0.5		
Premix	0.5		
Total	100		

[1] The calculated value of metabolizable energy and the measured value of other nutrients.

Table S2 Primers for quantitative real-time PCR.

Gene Name	Accession number	Primer sequence	Amplicon Size, bp
HAO1	XM_005688179.3	F: GGTGTGCGGAAGGGAACTGATG<br>R: CCAAGCCCCAAATGATCGGTCTC	88
LDHD	XM_018061713.1	F: AGCACCGTACACATCCTCCAG<br>R: GCCACGCAGCAGTTCAGC	110
MAT1A	XM_005699270.3	F: CTACGACGACTCCGCCAAGG<br>R: GCACCGACATCCTCTTCATTTCTG	123
GNMT	XM_018038609.1	F: GCTAACTGGATGACTCTGGACAAAG<br>R: CGGTGCTCGCTCTGGTCTC	125
AHCY	XM_018057737.1	F: GATTCTGGATGACGGTGGTGAC<br>R: AGATGCCTCGGATGCCTGAC	80
CCND1	Li, 2019[1]	F: GCCGAGGAGAACAAGCAGA<br>R: GAGGGTGGGTTGGAAATGAA	103
IVL	Li, 2019[1]	F: TGTGAGTCTGGTTGACCGAG<br>R: TGGGTATTGGTGGGAGGAGA	122
PCNA	XM_005688167.3	F: AGTGGCGTGAACCTACAGAGC<br>R: TACGGTCGCAGCGGTAAGTG	97
CDK4	XM_005680266.3	F: CCACCAGGCTTGCCAGTAGAG<br>R: CCACCACTTGTCACCAGAATGTTC	131
CDC42	XM_018055357.1	F: TAAGCAGAAGCCTATCACTCCAGAG<br>R: GCAGAGCACTCCACATACTTGAC	81
TGF-β	NM_001314142.1	F: TGACCCACAGAGAGGAAATAGAGG<br>R: ATGTCCACTTGAAGCGTGTTATCC	85
BMP4	NM_001285646.1	F: CGACCACCTCAACTCAACCAAC<br>R: CAGAACCACCTTGTCATACTCATCC	145
WNT10B	Liu et al., 2021[2]	F: TGCTCACAACCGCAACTC<br>R: GGTCTCGCTCGCAGAAG	107
β-catenin	Liu et al., 2021[2]	F: GACCACAAGCAGAGTGCT<br>R: TGTCAGGTGAAGTCCTAAA	100
GSK-3β	XM_018042977.1	F: GCCCAGAACCACCTCCTTTGC<br>R: ACCTTGCTGCCATCTTTGTCTCTG	99
LEF1	Ma et al., 2015[3]	F: CCATCCTCTCACCCCTCTCA<br>R: AAATCCAGCCAAGAGGTGGG	191
β-actin	Gong et al., 2022[4]	F: GGCAGGTCATCACCATCGG<br>R: CGTGTTGGCGTAGAGGTCTTT	158

[1]Li Mengmeng, Effects of methionie, cysteine and melatonin on proliferation of inner mongolia cashmere goat fibroblasts. Hohhot, Inner mongolia Agriculture University, 2019.

[2]Liu J, Mu Q, Liu Z, et al. Melatonin Regulates the Periodic Growth of Cashmere by Upregulating the Expression of Wnt10b and β-catenin in Inner Mongolia Cashmere Goats[J]. Frontiers in Genetics, 2021, 12: 665834.

[3]Ma sen, Zhang enping, Chen yulin, Yan hailong. Expression analysis of genes within wnt/β-catenin signaling in goat cashmere degeneration and pigmentation. Acta Ecologiae Animalis Domastici, 2015, 36(01):12-17.

[4]Gong G, Fan Y, Zhang Y, et al. The regulation mechanism of different hair types in inner Mongolia cashmere goat based on PI3K-AKT pathway and FGF21. Journal of Animal Science, 2022, 100(11): skac292.

Table S3: Effects of 2-Hydroxy-4-(methylthio)butanoic acid isopropyl ester (HMBi) supplementation on cashmere performance of goats.

	Group			
Items	CON	HMBi	SEM	*P* value
Cashmere length (mm)	20.88	23.59	0.382	<0.001
Cashmere diameter (μm)	17.66	16.37	0.331	0.046
average daily growth rate of cashmere (mm/d)	0.35	0.39	0.006	<0.001

Figure S1 The number of significantly differentially expressed genes (DEGs) in the HMBi group compared with the CON group. CON, the control group; HMBi, 2-hydroxy-4-(methylthio) butanoic acid isopropyl ester group.

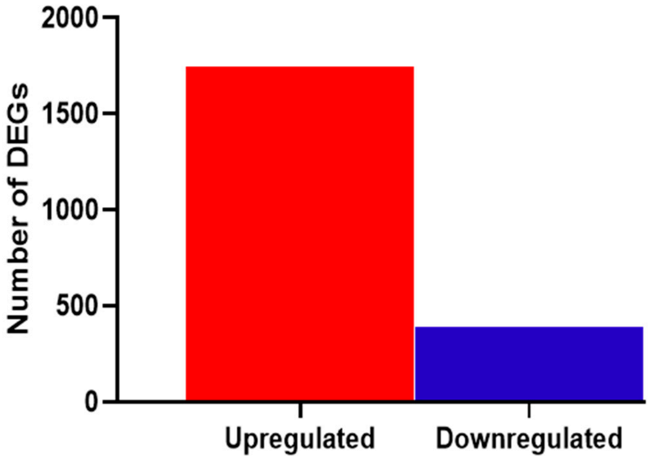

Figure S2 The enrichment analysis of GO terms identified from transcriptome in the skin tissue of goats.

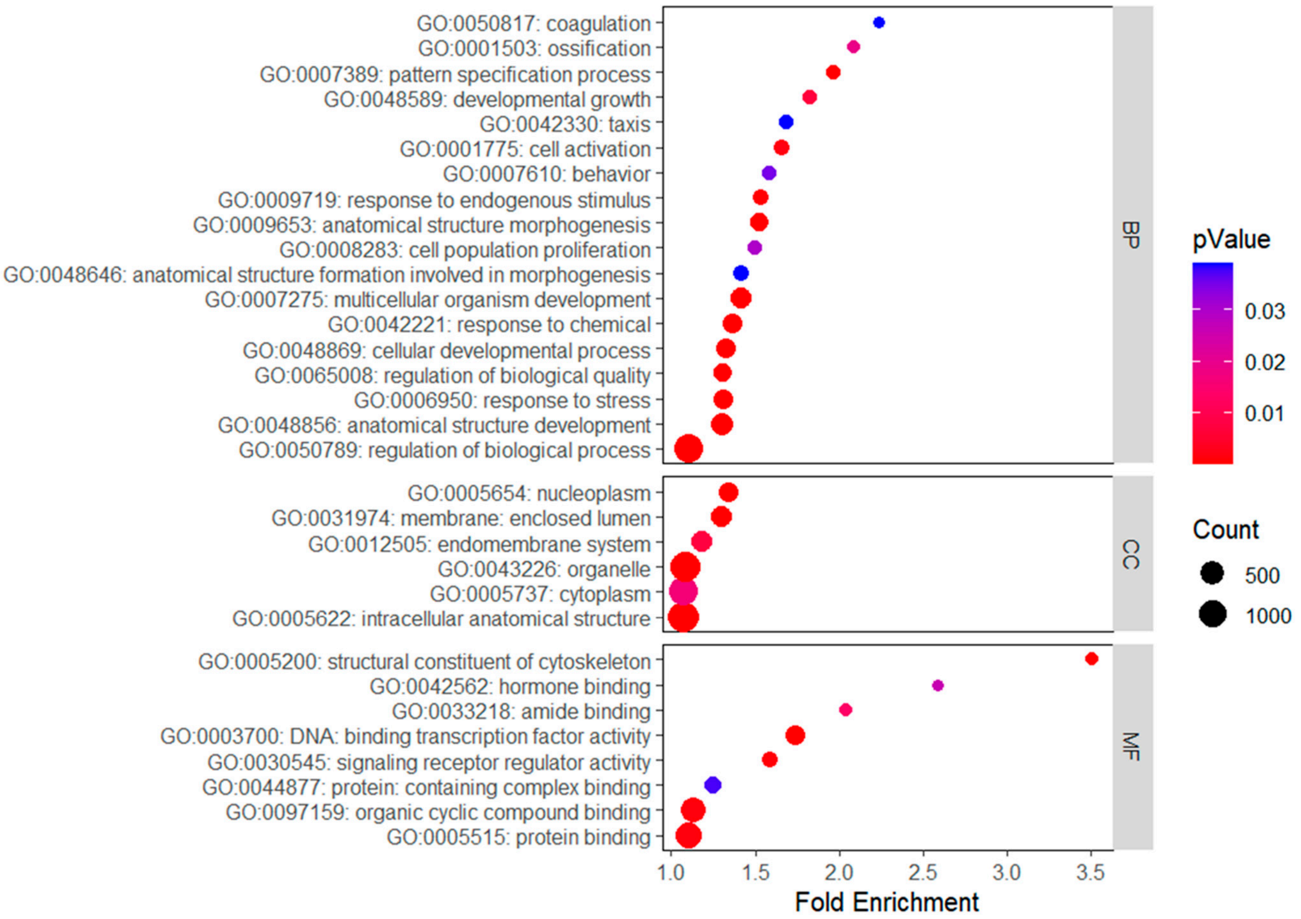

Supplement: Supplementary file 1 [file ijms-26-01663-s001.zip › ijms-3417840-supplementary.pdf]
